# Foucault’s Social, Community, and Cultural Psychology

**DOI:** 10.1007/s12124-024-09878-0

**Published:** 2025-01-14

**Authors:** Line Joranger

**Affiliations:** University of South-Eastern Norway,

**Keywords:** Psychological foundation, Interdisciplinary psychology, Critical psychology, 1954 foucault, User involvement, Contemporary social, Community, And cultural psychology, Environment

## Abstract

In today’s debate about a user oriented humanistic turn in the field of mental health care, the early Foucault is once again relevant. In his works from 1954 Foucault shows that the root of understanding mental phenomena is not to be found in universal medical concepts and methods, but in the reflection on lived experiences and in the human being itself. In accordance with contemporary social, community, and cultural psychologists, such as Brinkmann, Kinderman and Prilleltensky, Foucault is critical to the psychology’s medical foundations. Instead of focusing on medical and physiological matters he suggests that psychology, as a discipline, should be more user oriented, and focus more on the human being itself, and on social and cultural contexts.

After a century of trying to develop an objective and empirical science of mental phenomena, there is still no agreement on how to explain them (Joranger, 2015). Through the French historian of ideas and power analytic Michel Foucault’s, 1954works I want to show why the idea of an objective and empirical science of psychology is hard to reach. Through his 1954 works, Foucault reveals how mental phenomena are affected by unstable variables, such as cultural ideas, social contexts, and individual experiences.

Foucault has not always been anti-psychological, post-structuralist, or anti-humanist, as many seem to believe (Danziger, 1990; Han, 2002; Rajchman, 1985; Rose, 1990). In 1954, he declared a belief in a user oriented humanistic psychology. Unlike Foucault’s more (post)structuralist and anti-psychiatric texts from the 60s and 70s, which was later translated into English (Foucault, 1973, 1979, 1982, 1994), his earliest academic texts do not problematize whether there are psychological truths or not, or whether the subject exist or not. In line with many of today’s cultural and community psychologists, such as Peter Kinderman (Kinderman, 2014a, b, 2019), Svend Brinkmann (Brinkmann, 2017, 2020b, 2021) and Isaak Prilleltensky (Prilleltensky, 2008, 2012; Prilleltensky & Prilleltensky, 2021; Prilleltensky et al., 2023), he rather exhibits a unique will to understand human experiences and lived life as a source to psychological insight, as well as the value of social relationships.

In accordance with Kinderman, Brinkmann and Prilleltensky, Foucault in his 1954 works connects the human mind and psyche to a broader social, cultural, economic, political, and environmental influence to promote positive change, health, and empowerment, at individual and systemic levels. In his earliest works, Foucault believes that psychology, as a discipline, could have an important function in improving people’s wellbeing and challenges in everyday life, if it only could manage to establish itself as an independent discipline, different from the more quantitative sciences, such as biology, physiology or medicine. Foucault’s, 1954 works did not develop in a cultural or intellectual vacuum. His ideas draw on influential colleagues who suggested that a person’s personality are shaped in the interaction between experiences, nature, biology, history, and sociocultural contexts (see e.g. Canguilhem, 1988; Canguilhem, 1989; Viktor von Weizsäcker, 1933, 1940, 1951).

The objective of the article is to show that Foucault’s texts from 1954 still have relevance for today’s debate about the foundations of psychology, and how it should be used to approach mental challenges, user involvement, and wellbeing.

In 1954, Foucault wrote two texts concerning the restriction and development of modern psychology. He reflects upon mental illnesses and the nature of human mind, and the importance of understanding people’s everyday life and lived experiences. The first text was an introductory to the French translation of the German psychologist Ludwig Binswanger’s existentialist and phenomenological essay *Dream and Existence* (Traum und Existenz) (Binswanger, 1930), or Le Rêve et l’Existence, as it came to be called in the French 1954 edition (see Foucault, 2001). The second text was the book *Mental Illness and Personality* (Maladie mentale et personalité) (Foucault, 1954). The book has not been translated into other languages, I therefore refer to and translate from the original French 1954 edition. Both Foucault’s, 1954 texts problematise the concepts, methods and truths that exist in modern psychological praxis. Through his texts, Foucault aimed to show that the root of understanding psychological phenomena is not to be found in universal medical concepts, but in the reflection on the human life itself and on sociocultural contexts.

## The Physical and Psychological

In *Mental Illness and Personality* (1954), Foucault starts by reflecting on the question: What is the connection between the psychological theory and the somatic theory when it comes to illness? Diving into the history of psychology by analysing psychological theories and approaches, he finds that modern psychological theories and practices through history have used concepts and methods from the somatic area, and thereby reduced the understanding of human psyche to concerning brain, physiological structures and biological phenomena (Foucault, 1954, pp. 1–17). Foucault’s scepticism toward the use of medical concepts to explain human behaviour and experiences, has its background in a German French intellectual environment consisting of critical epistemologists and psychologists from the early nineteenth centuries. Among the most influential we find the French philosopher and epistemologist, Georges Canguilhem (1988, 1989). Canguilhem was Foucault’s supervisor on his doctoral thesis, ***Madness and Civilization; A History of Insanity in the Age of Reason***(Foucault, 1965). He spent his academic life to reflect on, and criticizing, what he believed to be an uncritical importation of biological concepts into the social arena. He challenged contemporary understanding of “normality” and “pathology” and how a psychiatrist should approach a mentally ill person (1988, 1989). Another influential thinker who was known to Foucault, was the German physician and physiologist Viktor von Weizsäcker (1933, 1940, 1956). According to von Weizsäcker a human being cannot be reduced to the categories of logics, to the mathematical formulations of physics and chemistry, that is, to the so-called exact sciences (De Luca Picione, 2024a, b) In line with these critical thinkers, Foucault suggests in his 1954 works that one instead of focusing on physiological matters, the psychology, as a discipline, should focus on the way people live and understand their life, and on social and cultural contexts.

Foucault believes biology and psychiatric medicine has played a decisive role in how contemporary psychologists deal with mental problems. In order to legitimize its scientific status, he thinks that today’s mental healthcare have chosen to adapt the medical diagnosis model both to understand and to explain mental phenomena and difficulties. Sixty and seventy years later, the same critical reflections and questions concerning modern psychology are hotly debated by scholars all over the world. The Danish professor and psychologist Svend Brinkmann, has in several essays, books, and articles dealt with the same questions, that is, the foundation of modern psychology. In the article *Psychology as a Science of Life*, he states that:

Psychologists, philosophers, and other human scientists are rightly eager to debate the nature of the mental, which quickly becomes a discussion about the mind as a noun that refers to an entity (with all the problems that follow from such reification), but they have been less eager to discuss the nature of life as such and ask the question: What do we mean when we talk about mental life? (…) Not only life in its biological manifestations, of course, as when we talk about someone “being alive” rather than dead (although this is certainly also significant), but life as a kind of conduct, as when we talk about someone “living a life.” (Brinkmann, 2020b, p. 4).

According to the English professor of clinical psychology, Peter Kinderman (2014), the biggest problem with the introduction of the medical “disease model” in the mental health care field is that the treatment of people’s stress and anxiety rests heavily on diagnoses without taking other conditions into account. In the book *A Prescription for Psychiatry: Why We Need a Whole New Approach to Mental Health and Wellbeing* he writes: “We must stop regarding people’s distress as merely the symptom of diagnosable illnesses’ and instead develop a more appropriate system of describing and defining people’s emotional problems.” (Kinderman, 2014a, p. 48).

In line with Brinkmann’s and Kinderman’s critical voices, the Argentinian professor and community psychologist, Isaak Prilleltensky (2021), is sceptical to the one dimensional and medical focus in current psychology. He highlights the importance of an integrated psychology where people’s wellbeing is seen in relation to feelings, experiences, culture, fairness and mattering. According to Prilleltensky et al. (2023, p. 203):

Like fairness and wellness, *mattering confers benefits upon individuals*,* relationships*,* and entire communities*. As shown above, feeling valued and having opportunities to add value make the individual feel like he or she matters. People who feel that they matter are more likely to contribute to the well-being of others and the community at large (…). Groups react intensely to feelings of being devalued, as demonstrated by the #BlackLivesMatter and #MeToo movements, to name only two.

Although Brinkmann, Kinderman and Prilleltensky represent different times and different nations than Foucault, they have in common that they move away from what within their contemporary mental health care represents a strong focus on diagnoses, medical models, and traditional treatment. Like Foucault, Brinkmann, Kinderman and Prilleltensky want a shift in focus – from a focus on medical models and concepts to a focus on the human itself, on lived life, and on social and cultural contexts.

By looking back at the origin of modern psychology, Foucault claims in his first book, that modern psychology does not have its origins in a unique and distinctive science of human being, but in the classic medicine’s symptomatology (observable signs) and nosology (disease descriptions) (1954, p. 2). He believes that the methodology on which modern psychology rests is based on a linguistic fraud. He writes:

Beyond the psychological and somatic theory of disease, there is a general and abstract understanding of disease that governs them both by imposing the same concepts on them and thus a series of prejudices that direct them to the same methods and thus a series of postulates. (Foucault, 1954, p. 2).

Foucault does not ignore that this ensures a unity that was previously lacking between the somatic and psychological disciplines but points out that it also represents a euphoria that displaces another reality; the impossibility of using the same methods and concepts in the psychological area as in the physical if the aim is to understand human beings. “I will try to show that the origin of the psychological illness cannot be sought in some metapathology, but in a reflection on human beings itself” (Foucault, 1954, p. 2).

In the same manner, Brinkmann claims: “When the subject matter is human mental life, it might be better to live with conceptual confusion than obtaining consensus on a misguided foundation (Brinkmann, 2020a, p. 589). He continues:

In order to understand human life, one should also study the science and practices through which people strive to understand their lives. Psychology should be inherently reflexive. The problem with a foundational program (…) is that it seeks to close down this reflexivity. It sees theoretical polyphony as a problem to be overcome so we can create a steady building, whereas the very fact that theoretical polyphony is possible should be the starting point for a general psychology that is aware of intentionality and normativity. (Brinkmann, 2020a, pp. 594–595)

Like Brinkmann, Foucault in his first book challenges the contemporary psychological model for not understanding its aim, that is, to understand human being such as they live and experiences, and for not understanding what separate it from other sciences, such as physic and medicine.

## Psychology as a Theoretical Polyphony

In his 1954 book, Foucault highlights three conditions which he believes confirm why we cannot use the same methods and concepts in psychological treatment as in the somatic or physical treatment. Firstly, he believes that psychology can never offer psychiatry what physiology can offer somatic: that is, a concrete analytical tool which, with its causal calculation methods, makes it possible to see and isolate the functional connections between the damaged (organic) area and the other healthy organs (Foucault, 1954, p. 13). He points out that our psychic life includes a completely different organization between cause and effect than our physical body. Psychic life includes a phenomenological understanding or a focus on “meaningful units of behaviour” (“l’unité significat”), which manifests itself in dreams, crime, unmotivated action sand free associations. Because the psychic life carries within it the person’s past experiences, the person’s attitudes and lifestyle, it cannot possibly, according to Foucault, use the same universal concepts and methods that the somatic area uses.

Secondly, unlike the somatic field, Foucault believes that psychology never can fully distinguish between normal and morbid functions (Foucault, 1954, p. 13). Psychology’s complex personality analyses depend, according to Foucault, on qualitative assessments, such as judgement, intuition and interpretation. These are assessments that allow for all possible mistakes, which makes it difficult to distinguish between the normal and the morbid. To explain what he means, Foucault highlights an example from recent research at the time which shows that different schizophrenic character traits occur in both normal and pathological personalities. One can therefore not conclude hundred prosect, according to Foucault, that these specific character traits belong to a purely morbid dimension.

In the third and last relationship, Foucault emphasizes the impossibility of understanding the sick person’s own perceptions of reality with the help of biological concepts or an unchanging notion of what is sick and healthy psychic phenomena (Foucault, 1954, p. 15). Like many of today’s cultural and social psychologists, such as Brinkmann, Kinderman and Prilleltensky, he claims that a person’s experience of reality must be understood from the perspective of the patient’s particular social and cultural environmental conditions. He believes that the Insanity Act of 1838 is an example of a significant environmental condition that came to influence the living situation of the mentally ill. The law, according to Foucault, gave doctors absolute authority over the sick, which contributed to a legal and moral disempowerment by transferring the patient’s rights to a chosen guardian. This, he believes, set the stage for an alienating environment which, among other things, created the despairing hysteric.

The fact that the hysteric in the 1950s was about to disappear was, according to Foucault, not due to physiological reasons, but of environmental reasons, that is, environmental changes. The socio-cultural environment inside and outside the hospital had become less rigid, and the wherein larger focus on women’s rights and wellbeing. This shows, Foucault believes, that the dialectic between the individual and the environmental does not proceed in the same way within the physiological area as within the psychological area. Because the environment has such a great effect on the human mind, psychologists, in addition to conducting therapy, should facilitate the patient’s daily environment by changing the social conditions that may cause the illness ((Foucault, 1954, p. 16).

In *Mental Illness and Personality*, Foucault, like Brinkman, Kinderman, and Prilleltensky urges that within current psychology one must complement the patient’s own experiences with a socio-cultural explanatory model and a polyphony of theories and understandings. He encourages psychologists to approach their patients or human beings with multiple perspectives and theories. In line with Prilleltensky’s theories (Prilleltensky, 2012; Prilleltensky & Prilleltensky, 2021; Prilleltensky et al., 2023), one should at the same time see the personal, physiological and socio-cultural aspects of human life. Like Prilleltensky, he believes that these multiple perspectives make it possible to explain the connection between psychological problems and socio-cultural environmental conditions that effect people at any given time.

In *How People Matter*, Prilleltensky and Prilleltensky (2021) discuss healthy and unhealthy cultures and how cultures, liberty, and fairness affect people’s wellbeing and mental state. They show how some cultures foster mattering for everyone, while others erode it through division and inequality. They write:

The sense of mattering we experience in communities is a function of the personality of their inhabitants and the norms of that particular culture. Cultures that promote inclusion nudge individuals to be fair, caring, and compassionate. Cultures that ignore power differentials pretend that we are all the same and that we all experience identical privilege. Cultures that infringe on people’s rights to a psychological safe environment enable bullying Personality and culture interact all the time (2021, pp. 232–233).

In his 1954 book, Foucault points to a time-typical social and cultural structure in Western democracies that depends on a population that is highly competent in managing complex social contexts, while at the same time being able to communicate their needs and opinions. Mental problems, he believes, arise in this environmental context when people with less social personality traits are pressured to adapt to the demand for social participation and communication that they (by nature) are not equipped to handle. For this group of people, the environment’s demands for communication and social participation can lead to psychological stress reactions, which also manifest themselves in physiological reactions. In this case, it is important to understand that it is not the biological reactions that have led to psychological stress, but the opposite. Foucault’s considerations appear again in Kinderman’s (2014a, p. 1) ideas:

We must move away from the ‘disease model’, which assumes that emotional distress is merely a symptom of biological illness, and instead embrace a psychological and social approach to mental health and well-being that recognizes our essential and shared humanity.

To Foucault, Kinderman and Prilleltensky, in the age of diagnosis and method, there is a tendency to neglect the cultural and context perspective in favour of the medical and natural science perspective. They believe that an understanding of psychological phenomena also implies an understanding of social and cultural phenomena.

## The User Perspective – the Patient’s Experience of Illness

Brinkmann, Prilleltensky, Kinderman and Foucault (1954) believe that the mental health field has a high degree of expert management, with a clear professional and knowledge hierarchy. This, they believe, can override the user’s perspective and the patient’s own experience of illness. To Prilleltensky (2008, p. 120), the idea of internalized social prescriptions has direct implications for the self-perception of people with psychological problems.

Although coercion has not disappeared from the treatment of the mentally ill, we have today treatment methods characterized by kindness and compassion. However humanitarian, this turn is not without side effects, for it shifts responsibility for problems and solutions inward. In the absence of apparent coercion, and in the presence of overt caring, there is nobody but oneself to blame for difficulties and lack of progress. (…) Great care should be exercised in working with community members to ensure that we do not, however innocently, contribute to practices and discourses of oppression and conformity. (…) Hence, we need to reflect on what is our role vis a` vis clients and community partners who engage in oppression of others. Not an easy question, for we tend to medicalize the problem and avoid the difficult moral dilemmas. (Prilleltensky, 2008, pp. 120–121)

Prilleltensky believes that today’s psychology has to a small extent challenged the existing medical knowledge paradigm, but rather maintained it to preserve its own position, which he believes goes beyond the user’s perspective. Foucault (1954) also calls for a user voice within the contemporary psychological and psychiatric treatment system. He believes that it is not the case that the doctor or psychologist unequivocally stands on the side of health, as the one who alone possesses the truth and all the knowledge about the disease. Nor is it the case that the patient unequivocally stands on the side of the disease, as the one who is ignorant of everything that concerns him or herself and their existence (Foucault, 1954, p. 56).

Research on schizophrenics, such as that carried out by the German psychiatrist Jakob Wyrsch in the paper, *Die Person des Schizophrenen*, translated into Frensh as, *La personne du schizophène* (1956), show, according to Foucault, that even people with severe psychosis can have a suggestive recognition and a diffuse perception of the morbid framework in which the pathological themes stand out (Foucault, 1954, p. 57).

Foucault (1954, p. 58) recalls that the patient, in several forms of obsessive-compulsive disorder and in several forms of paranoia and schizophrenia, can recognize how the course of the illness is paradoxically linked to his/hers concerned personality. In other words, it is not as if the unique awareness of illness is erased, even if it has many objective and evident illness characteristics. For example, patients never confuse their doctor’s voice with the hallucinatory voice that constantly haunts them. This even though the doctor also appears as a persecutor to the sick person (1954, p. 59). In this theatrical perception of reality, the awareness of illness manifests itself as an awareness of another reality. By coming to terms with the fact that it is only he/she, and not the others, who hears voices, the mentally ill person recognizes the alien and painful peculiarity of his/her universe. By coming to terms with his/her two worlds in this way, and by adapting to the first world as well as the second, he/she expresses a specific awareness of his own illness as the background for his/her behavior (p. 59). Even in the extreme form of schizophrenia, Foucault believes that the patient has an oceanic sense of his/her illness. The patient’s universe is thus never an absolute, it always unfolds with an inherent double reference between the normal and the pathological, the familiar and the foreign, the specific and the general, the awake and the dreaming.

## The Boundary Breaking Properties of the Human Mind

Foucault’s notions of the human mind’s boundary breaking properties challenge the medical methods and the diagnostic concepts. Kinderman’s ideas about the human mind do as well. He writes:

Traditional psychiatric diagnoses are arbitrary and invalid, and do not map onto biological processes or describe real illnesses. They are also circular concepts, attempting to explain human behavior merely by labeling it as pathological. This reinforces a reductionist biological view of mental health and well-being and encourages discrimination and the use of inappropriate medical treatments. (Kinderman, 2014a, p. 48).

In an attempt to avoid any form of medical reductionism, Foucault (2001) in his second 1954 work, the Introduction to Binswanger’s essay *Dream and Existence*, approaches the human mind through a poetic repertoire of concepts. The mind represents here the human imaginary world, which symbolizes the ability to produce quite accurate images of things that you want, or don’t want, to happen. With the help of our imagination and the ability to remember, the human psyche can also imagine things that have already happened. This applies, for example, to the ability to imagine that that the person we love still is sitting next to us on the sofa, even if this person has moved out, several years ago. Foucault relates the human imagination and its ability to remember things in the past and predicted things in the future, to the transcendent nature of the human mind. Our dreams and our imaginations let us experience ourselves and our environment in the past, in the present, and in the future. Understanding this kind of psychological phenomena requires, according to Foucault, a basic analysis of the human mind that is neither logical-philosophical nor medical-psychological. It requires a form of analysis whose concepts, principles, and methods alone are derived from the world of unique human experiences.

In the 1954 introduction text (see Foucault, 2001), dream experiences are related to the real content of human life, which is to live and experience oneself without trying to reduce it, or adapt it, to any form of cause-and-effect analysis. In this text, the human’s ability to imagine, is not about another way of experiencing a ‘new’ world, but about a radical way of experiencing one’s own world, such as it is lived and understood. In the imaginative dream experiences, everything is centred around lived life. It is a mirror and a window to understand life, such as it is lived. The privileged status of the dream experience thus encompasses an entire anthropology of imagination which necessitates a new way of finding out how the relationship between meaning and conceptual expression manifests itself within everyone (see Foucault, 2001, p. 133).

According to Foucault (see 2001), poetry is the genre that can best express the anthropology of the human imagination. He believes that great poetry refuses to reduce its expressions to standardized images, such as medical concepts and models. Great poets will never be able to fulfil their desire to express human life by using static images of reality, because the freedom of imagination imposes itself on him/her, as a calling. In its true poetic function, the performance meditates on identity, that is, who one is and who one wants to become. In contrast to the free nature of the performance, the morbid imagination - the crude hallucination where the performance is totally imprisoned in a fantasy image of reality - stands. This occurs when the patient finds the free movement of existence crushed by an image that immobilizes the imagination. The function of the imagination, which is to experience and create oneself by crushing such static fantasy images, has here collapsed, and the patient is left to an unknown reality.

## Critical to Psychological Positivism

Foucault’s 1954 texts were created in a period before Foucault became the Foucault that most of us know and have read, that is, the anti-psychologist and the postmodernist. I believe that without understanding his 1954 works, we will never fully comprehend Foucault’s later, more ‘poststructuralist’ works on power, discourses, and discipline. I believe that those who take the time to read Foucault’s 1954 works will have to change their stereotypical perceptions of Foucault as one who proclaims the death of “the subject” once and for all, and at the same time understand that Foucault fought a lifelong struggle to preserve the voice of the oppressed subject.

Brinkmann, Kinderman, Prilleltensky and Foucault’s critical view of modern medical psychology joins the 1950s positivism debate, represented by, among others, Popper (1959), Kuhn (1962) and Wittgenstein (1958). Like the positivism debate in the 1950s, it seems that Foucault, Kinderman, Brinkmann, and Prilleltensky want to develop a critical rationalism within their contemporary mental health care. This entails a reorientation both conceptually, methodologically and content-wise, by, among other things, doing ​​research without “standardized” preconditions (naive empiricism), and preconceived beliefs about outcomes. They all believe that psychological research goes far beyond objective research and that the human psyche has more border breaking sides than one empirically can demonstrate. The study of the human being must align with how people live their lives in specific cultures and communities. To approach the human mind, one needs an interdisciplinary view that can capture life as it is experienced and navigated. Practical psychology thus does not belong to the medical concepts and disciplines, it both needs and deserves its own concepts and its own basic thinking.

My own point of view on this subject matter is that by focusing exclusively on the processes that go on within a person, rather than on the processes within which a person goes on, we misinterpret how experiences, culture and community effect a person’s perceptions and cognitions. In the study of human behaviour, there are external facts that we can explain logically or physically. What we cannot explain is what these facts are, independent of perceptual standpoints and cultural contexts. Because we always already are defined by the world or by our sociocultural surroundings, this indicates that an independently existing person does not go into an independently existing world of which the person has an independent experience or illness. Scientists who study mental phenomena will always have to define their ideas in relation to someone or something already defined, a mindset, a rule, a theory, a culture, an environment, or a position.

## Data Availability

No datasets were generated or analysed during the current study.

## References

[CR1] Binswanger, L. (1930). *Traum und Existenz*. Girsberger. Sonderdr. ed.

[CR2] Brinkmann, S. (2017). *Persons and their minds: Towards an integrative theory of the mediated mind* (1st ed.). Routledge. 10.4324/9781315623658

[CR3] Brinkmann, S. (2020a). Moving on our feet: For a nomadic psychology. *Integr Psychol Behav Sci*, *54*(3), 589–596. 10.1007/s12124-020-09529-0 32279199 10.1007/s12124-020-09529-0

[CR4] Brinkmann, S. (2020b). Psychology as a science of life. *Theory & Psychology*, *30*(1), 3–17. 10.1177/0959354319889186

[CR5] Brinkmann, S. (2021). Resources and resonance: Notes on patiency as world relation. *Culture & Psychology*, *27*(4), 562–576. 10.1177/1354067X211017306

[CR6] Canguilhem, G. (1988). *Ideology and rationality in the history of the Life sciences*. MIT Press.

[CR7] Canguilhem, G. (1989). *The normal and the pathological*. Zone Books.

[CR8] Danziger, K. (1990). *Constructing the subject: Historical origins of Psychological Research*. Cambridge University Press.

[CR9] De Picione, L., R (2024a). Viktor Von Weizsäcker’s Gestaltkreis Theory. The dynamic and subjective relationship of Perception and Movement. *Integr Psychol Behav Sci*, *58*(1), 35–45. 10.1007/s12124-023-09787-8 37273098 10.1007/s12124-023-09787-8

[CR10] De Picione, L., R (2024b). Viktor Von Weizsäcker’s notion of Pathic: Affective liminality, modalization of experience and illness. *Integr Psychol Behav Sci*, *58*(1), 46–58. 10.1007/s12124-023-09791-y 37347408 10.1007/s12124-023-09791-y

[CR11] Foucault, M. (1954). *Maladie Mentale et personnalité*. Presses Universitaires de France.

[CR12] Foucault, M. (1965). *Madness and civilization; a history of insanity in the age of reason*. Pantheon Books.

[CR13] Foucault, M. (1973). *Madness and civilization; a history of insanity in the age of reason*. Vintage Books.

[CR14] Foucault, M. (1979). *Discipline and punish: The birth of the prison*. Vintage Books.

[CR15] Foucault, M. (1982). *The archaeology of knowledge; and, the discourse on language* (1st Pantheon pbk. ed.). New York: Pantheon.

[CR16] Foucault, M. (1994). *The order of things; an archaeology of the human sciences*. Vintage Books.

[CR17] Foucault, M. (2001). Introduction: Le Rêve et l’Existence (J. Verdeaux, Trans). In D. Defert, F. Ewald, & J. Lagrange (Eds.), *Dits et écrits, 1954–1976* (Vol. 1, pp. 93–147). Gallimard.

[CR18] Han, B. (2002). *Foucault’s critical project: between the transcendental and the historical* (1st U.S. edition. ed.). Stanford, Calif.: Stanford University Press.

[CR19] Joranger, L. (2015). *Subjectivity as science and experience: An existential-phenomenological and historical approach to subjectivity, objectivity, and psychology* (Vol. 543no.). Department of Psychology, Faculty of Social Sciences, University of Oslo.

[CR20] Kinderman, P. (2014a). *A prescription for psychiatry: Why we need a whole new approach to mental health and wellbeing*. Palgrave Macmillan.

[CR21] Kinderman, P. (2014b). The role of the psychologist in social change. *International Journal of Social Psychiatry*, *60*(4), 403–405. 10.1177/0020764013491741 23788437 10.1177/0020764013491741

[CR22] Kinderman, P. (2019). *A manifesto for mental health: why we need a revolution in mental health care*(1st 2019. ed.).

[CR23] Kuhn, T. S. (1962). *The structure of scientific revolutions*. University of Chicago Press.

[CR24] Popper, K. R. (1959). *The logic of scientific discovery*. Hutchinson.

[CR25] Prilleltensky, I. (2008). The role of power in wellness, oppression, and liberation: The promise of psychopolitical validity. *J Community Psychol*, *36*(2), 116–136. 10.1002/jcop.20225

[CR26] Prilleltensky, I. (2012). Wellness as Fairness. *American Journal of Community Psychology*, *49*(1–2), 1–21. 10.1007/s10464-011-9448-8 21643926 10.1007/s10464-011-9448-8

[CR27] Prilleltensky, I., & Prilleltensky, O. (2021). *How people matter: Why it affects health, happiness, love, work, and society*. Cambridge University Press.

[CR28] Prilleltensky, I., Scarpa, M., Ness, O., & Di Martino, S. (2023). Mattering, wellness, and fairness: Psychosocial goods for the common good. *American Journey of Orthopsychiatry*, *93*(3), 198–210. 10.1037/ort0000668 10.1037/ort0000668 37023268

[CR29] Rajchman, J. (1985). *Michel Foucault: The freedom of philosophy*. Columbia University.

[CR30] Rose, N. S. (1990). *Governing the soul: The shaping of the private self*. Routledge.

[CR31] von Weizsäcker, V. (1933). Der Gestaltkreis, dargestellt als psychophysiologische Analyse Des Optischen Drehversuches. *Pflüger’s Archiv für die Gesamte Physiologie Des Menschen Und Der Tiere*, *231*(1), 630–661. 10.1007/BF01754578

[CR32] von Weizsäcker, V. (1940). *Der Gestaltkreis: theorie der Einheit von Wahrnehmen und Bewegen*. Leipzig.

[CR33] von Weizsäcker, V. (1951). *Der kranke Mensch: eine Einführung in die medizinische Anthropologie*. Stuttgart.

[CR34] von Weizsäcker, V. (1956). *Pathosophie*. Vandenhoeck & Ruprecht.

[CR35] Wittgenstein, L. (1958). *Philosophical investigations (G. E. M. Anscombe Ed* (2nd ed.). Basil Blackwell.

[CR36] Wyrsche, J. (1956). *La Personne Du schizophène: étude clinique, psychologique, anthropophénoménologique*. Trans.). Presses universitaires de France. J. Verdeaux.

